# Can Tailoring the Polyamide Active Layer of the Desalination Membranes Ease the Recycling of End-of-Life Membranes for Seawater Pretreatment Applications

**DOI:** 10.3390/polym18172153

**Published:** 2026-09-03

**Authors:** Abdul Waheed, Umair Baig

**Affiliations:** Interdisciplinary Research Center for Membranes and Water Security, King Fahd University of Petroleum & Minerals, Dhahran 31261, Saudi Arabia; abdul.waheed@kfupm.edu.sa

**Keywords:** end of life membranes, desalination, sodium hypochlorite, polyamide membrane reuse, wastewater/seawater treatment

## Abstract

Millions of End-of-Life (EoL) reverse osmosis (RO) membranes are discarded each year by desalination facilities worldwide. This poses a significant environmental challenge, as large amounts of solid waste are often managed through landfilling or incineration. Recycling EoL RO membranes for reuse as nanofiltration (NF) or ultrafiltration (UF) membranes is an attractive pathway for the effective management of solid waste. However, the commonly adopted route to degrade the polyamide active layer of the EoL membranes for recycling and reuse requires high-intensity exposure of the membranes to the NaOCl solution. There is a need to develop a route to make membranes more responsive to the low dosage of NaOCl for recycling and reuse of the membranes. The current study explored that tailoring of the membrane active layer could generate a more responsive polyamide membrane to a mild concentration of NaOCl. This was achieved by incorporating monobenzoyl chlorinated aromatic reactants 4-nitrobenzoyl chloride (4-NC) and 3,5-dinitrobenzoyl chloride (3,5-DNC) into the active layer of the membranes. A minor concentration of the 4-NC and 3,5-DNC was added to the trimesoyl chloride-containing organic phase during interfacial polymerization with m-phenylenediamine. Owing to the presence of only one reactive site in 4-NC and 3,5-DNC, the reaction with m-phenylenediamine can hinder crosslinking in certain regions of the m-phenylenediamine and trimesoyl chloride polyamide active layer. This limited number of uncross-linked regions not only increases the membrane permeability with reasonable salt rejection but also makes the membranes more responsive to chlorine. This was realized when the permeate flux was found to be 108, 114, and 200 L m^−2^ h^−1^ for the M1 (control), M2 (4-NC), and M3 (3,5-DNC) membranes, respectively, at a feed pressure of 30 bar using 100 ppm of NaOCl for 24 h. On the other hand, the rejection of NaCl was decreased from 97 to 9.6, 93 to 7.1, and 91 to 3.6% with an increase in contact time from 2 h to 24 h for M1, M2, and M3 membranes, respectively. This exposure intensity was significantly less than that commonly used in the literature. Furthermore, the recycled modified membrane, especially M3, showed reversible fouling when the membranes were tested using bovine serum albumin as a model foulant in seawater scenarios.

## 1. Introduction

The development of reverse osmosis (RO) membranes is pivotal to desalination facilities all over the globe [1]. These desalination facilities are the lifelines of the masses around the globe, especially in the most arid regions of the world [2]. The polyamide membranes, including RO and nanofiltration (NF) membranes, are prepared by interfacial polymerization (IP), which is a technique widely applied for tailoring the chemistry of the membrane’s active layer [3]. Hence, to keep these facilities running, an immense number of RO membrane elements are generated, and a huge number of End-of-Life (EoL) RO elements are discarded as well [4,5]. Even though the RO membranes have served humanity by providing water for different life activities, there is also a considerable environmental concern associated with the RO membranes [6,7,8,9]. These challenges are related to the production of the membranes, where different chemicals and polymers are used, which are mostly obtained by processing petrochemicals [10,11]. Therefore, the use and disposal of solvents cause environmental concern. Some studies have been conducted on green solvents for the fabrication of membranes to limit the environmental impact of the commonly used organic solvents for membrane fabrication [12,13]. However, the most pressing issue is the solid waste generated by EoL elements, which was estimated to be 2 million spiral wound RO modules by 2025 [14]. An estimation of the average weight of an 8-inch RO module to be between 13 and 15 Kg has proposed that around 30,000 t will be generated by 2025 [15]. Disposal and management of such an immense amount of EoL membrane modules is a significant challenge that has not been well explored. Generally, the RO modules are fabricated, imported/purchased, installed, used, and discarded. Hence, there is a need to come up with strategies to resolve the challenges associated with EoL membranes and make them reusable for applications such as wastewater treatment or seawater pretreatment.

Different approaches have been adopted to tackle the EoL membranes, such as landfill disposal and incineration [16]. However, landfill disposal and incineration are environmentally and economically unsafe [17,18]. One of the best ways to deal with the challenge of managing such an enormous amount of solid waste is to recycle the EoL membranes for application NF or seawater/wastewater treatment as ultrafiltration (UF) membranes. A recent study has shown that EoL seawater (SW) and brackish water (BW) RO membranes could be regenerated to the desired levels of degradation of the polyamide active layer of the membrane [19]. Both membrane SWRO and BWRO were treated with the free chlorine solution of sodium hypochlorite (NaOCl) of varying strengths and durations, resulting in partial and full degradation of the active layer. It was found that the permeability of the membranes was increased upon exposure to chlorination, with an obvious decrease in the selectivity where the recycled membrane behaved in an NF-like manner. However, it was observed that the commercial SWRO membrane resisted the attack of chlorine to a greater extent, requiring high dosage and extended duration of chlorine exposure to the membrane. The same group also reported the fact that recycling of the BWRO membrane to UF is far more feasible both environmentally and economically than converting a SWRO to UF [20]. Therefore, there is a need to come up with a novel strategy to tailor the active layer chemistry in such a way that it becomes sensitive to the attack of chlorine.

Another study reported the use of different oxidative agents, including KMnO_4_, H_2_O_2_, and NaOCl, for the recycling of the polyamide RO membranes [21]. It was found that a very high-exposure intensity of 300,000 ppm·h of NaOCl, and KMnO_4_ was effective in removing the polyamide active layer of the membrane. To reduce the need for a high concentration of the oxidative agents, such as KMnO_4_, a new strategy was proposed to couple the use of oxidative agents with ultrasonication [17]. This approach resulted in a 70-fold increase in the permeability of the membrane to be reused for microfiltration applications. These examples showed that the direct reuse of the RO membranes has huge potential with multiple advantages. These include advantages such as an avenue of circular economy, environmental sustainability, and innovative solutions for wastewater treatment and seawater pretreatment. However, most of the literature has focused on regenerating the commercial EoL membranes, which have a highly dense polyamide active layer, such as in the case of SWRO membranes [22]. Because the polyamide active layers are significantly dense with extensive crosslinking, making the degradation quite challenging, requiring high-exposure intensities and the presence of basic conditions [23]. The use of high-exposure intensities of oxidative agents is also potentially an environmentally unsafe practice [24]. Very little or no attention has been paid to tuning the membrane active layer in a manner that tailored membranes could be recycled or reused using low or mild dosages of the oxidative agents at the EoL of desalination membranes.

The current study explored for the first time the ease of degradation of the polyamide active layer to demonstrate the development of an environmentally less intense approach for recycling EoL RO membranes for reuse in pretreatment applications. To achieve this, the active layer of the polyamide membrane was tuned by incorporating minor quantities of the mono-benzoyl chlorinated reactive additives 4-nitrobenzoyl chloride (4-NC) and 3,5-dinitrobenzoyl chloride (3,5-DNC). These membranes were fabricated by adopting the procedure mentioned elsewhere [25], where the desalination potentials of the membranes have been thoroughly studied. The presence of one acid chloride group on the 4-NC and 3,5-DNC enables reaction with m-phenylenediamine during interfacial polymerization. The trimesoyl chloride has three acid chloride groups, which offer sites for extensive cross-linking upon reaction with m-phenylenediamine. However, the presence of minor amounts of 4-NC or 3,5-DNC in addition to trimesoyl chloride during interfacial polymerization with m-phenylenediamine could generate a limited number of linear plugs of polyamide chains in the otherwise cross-linked active layer. These regions can offer sites for easy breakdown of the polyamide active layer upon exposure to the chlorine solution. This was realized when the modified membranes showed more response to the chlorine compared to the control membrane, which was fabricated using only m-phenylenediamine and trimesoyl chloride. The modified membranes showed significant enhancement compared to the control membrane in permeability, with reduced rejection of salt upon exposure to mild concentrations of chlorine over a short period of time. When tested against bovine serum albumin (BSA) as a model proteinaceous foulant to mimic seawater feed, the modified membranes showed reversible fouling. This strategy highlighted a route to tailor the chemistry of the membrane in a manner that enables the use of the membrane for desalination and facile EoL recycling for reuse in seawater pretreatment and wastewater treatment.

## 2. Materials and Methods

### 2.1. Materials

Polysulfone (PSf, Udel 3500, Solvay), polyvinylpyrroldine (PVP; M.wt. = 10,000, Sigma), dimethlyacetamide (DMAc, Sigma, Germany), sodium dodecyl sulfate (SDS, Merck, Germany), 4-nitrobenzoyl chloride (4-NC, Sigma, China) and 3,5-dinitrobenzoyl chloride (3,5-DNC, Sigma, Czech Republic), sodium hypochloride (NaOCl, Sigma) and triethylamine (TEA, Sigma, USA), sodium chloride (NaCl, PanReac, AppliChem, Darmstadt, Germany), Bovine serum albumin (BSA, Sigma, USA).

### 2.2. Fabrication of Membranes

**Figure 1:**
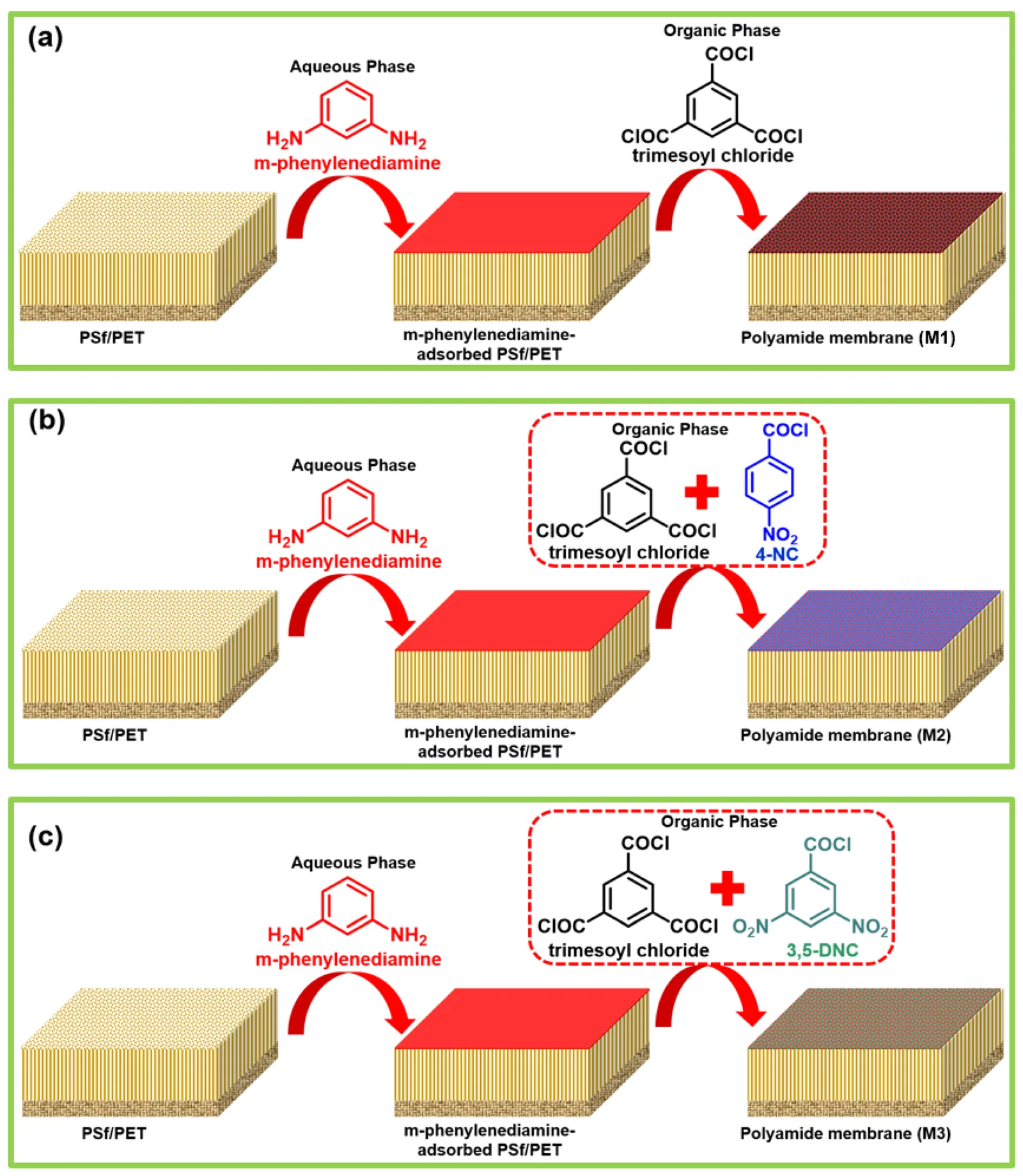
Schematic sketch for the fabrication of (**a**) M1, (**b**) M2, and (**c**) M3 membranes using interfacial polymerization.

The membranes were fabricated using a reported procedure [25]. In brief, the polysulfone supports were fabricated by wet-phase inversion. In brief, a PSf dope solution was prepared by dissolving 18 g of the dried PSf and 2.0 g of PVP in 80 g of DMAc, and the solution was heated at ~85 °C, and stirring was continued until a transparent solution was obtained. An appropriate amount of the PSf dope was cast on a non-woven fabric polyethylene terephthalate (PET) using a 200 µm doctor’s blade (custom made). Then it was dipped in a distilled water-containing coagulation bath for phase inversion, yielding PSf/PET support. The PSf/PET supports were dipped separately in two containers of aqueous solution containing m-phenylenediamine. Then the supports were removed from the m-phenylenediamine, and excess amine was removed by a rubber roller and membranes were allowed to dry visibly at room temperature. Then, the amine loaded supports were dipped in the organic phase of a specific composition. One of them was dipped in an organic phase containing 0.14 wt% trimesoyl chloride, having 0.01 wt% of 4-NC. The other amine-loaded polysulfone support was dipped in an organic phase containing 0.14 wt% trimesoyl chloride + 0.01 wt% of 3,5-DNC. The reaction was continued for ~60 s for completion of interfacial polymerization on the surface of polysulfone support. Hence, the obtained membranes were cured in an air-forced oven (SCI FINETECH, Korea) for a duration of ~20 min at 80 °C, leading to a cross-linked polyamide network. A control membrane was also prepared following the same route as used for developing the tailored membranes containing 4-NC and 3,5-DNC. The membranes were labelled as M1, M2, and M3, representing control, 4-NC, and 3,5-DNC, respectively. Schematic sketch for the fabrication of M1, M2, and M3 membranes is depicted in Figure 1.

### 2.3. Characterization of Membranes

Membranes were characterized using Fourier transform infrared spectroscopy (FTIR, Nicolet Is50, Thermo, Assembled in USA), scanning electron microscopy (SEM, EM-30AX, COXEM, Korea) coupled with energy-dispersive X-ray spectroscopy (EDX, OXFORD instruments) and elemental mapping, cross-sectional SEM, zeta potential analyzer (SurPass 3, Anton Par, Austria), and drop-shape analyzer (DSA25, KRUSS, Germany).

### 2.4. Performance Evaluation of the Membranes

The performance of the membranes was evaluated using the cross-flow filtration unit (High-Pressure Membrane Filtration Tester, BONA TYLG-19, Shandong Bona Group, China). All three membranes, M1, M2, and M3, were installed in parallel to each other in the cross-flow unit, and the feed was supplied by a high-pressure pump (China), and the temperature of the feed was maintained at ~22 ± 3 °C by a chiller (China) connected to a jacket surrounding the feed tank. The membranes were initially compacted using distilled water as feed for a duration of around 3 h at a constant pressure of 30 bar. After compacting the membranes, different experiments, such as the permeate flux and rejection of salts of the membranes before and after exposure to chlorine, were conducted. The permeate flux and salt rejections were measured by the following set of equations;

(1) $$J=\frac{V}{A\times t}$$

(2) $$R\left(\%\right)=\frac{C_{f}-C_{p}}{C_{f}}\times 100$$

In Equation (1), *J* is the permeate flux (L m^−2^ h^−1^), *V* is the volume (L) of the permeate generated by the membranes in a given time interval t (h) with an effective area of *A* (m^2^). In the case of rejection, Equation (2) represents C*_f_* and C*p* as the concentration of the salts in the feed and permeate samples, respectively, collected during the desalination experiments. Furthermore, the effect of increasing the concentration of NaOCl was also studied, where the membranes were dipped in NaOCl ranging from 10 to 100 ppm for a period of 2 h. In another experiment, the effect of increasing the duration of contact time of the membranes with NaOCl was also studied, where the concentration of NaOCl was kept constant at 100 ppm and the contact time was increased from 2 to 24 h. Upon optimizing the conditions of degradation of the polyamide active layer using 24 h and 100 ppm dosage of NaOCl, the recycled membranes were tested further. The effect of increasing feed pressure on the permeate flux was also measured where the feed pressure was varied from 2–10 bar. To judge the pretreatment/wastewater treatment potential of the membranes, the recycled membranes were tested using BSA as a proteinaceous foulant. The variations in the normalized flux were measured during this experiment for a duration of 14 h. Moreover, the membranes were cleaned by flushing the membranes with distilled water using the same crossflow system for 30 min at a constant feed pressure of 4 bar. Following cleaning, the recycled membranes were retested for BSA-containing NaCl feed for another 14 h at a pressure of 4 bar. During these experiments, the performance of membranes was further determined using the flux decline ratio (FDR) and flux recovery ratio (FRR).

## 3. Results and Discussion

**Figure 2:**
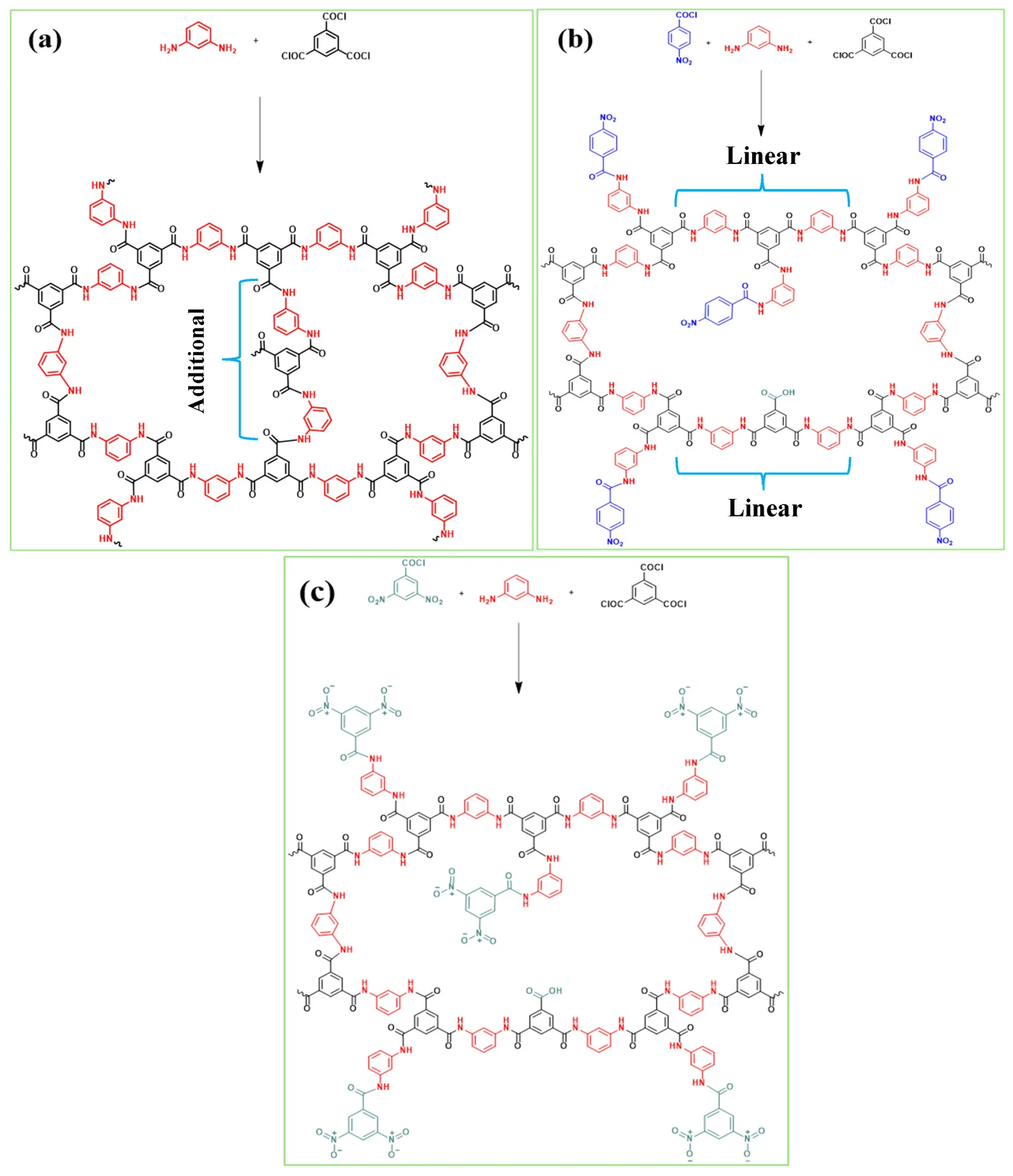
Possible structures of the polyamide active layers generated by interfacial polymerization reaction between the m-phenylenediamine and trimesoyl chloride (**a**), trimesoyl chloride + 4-NC (**b**), and trimesoyl chloride + 3,5-DNC (**c**).

Figure 2a shows the indicative structure of the polyamide active layer generated by the reaction of m-phenylenediamine and trimesoyl chloride during interfacial polymerization. Since m-phenylenediamine has two amino groups, it can cross-link the growing polyamide chains, resulting in a highly dense, cross-linked polyamide active layer responsible for the rejection of salts [26]. However, incorporating minor concentrations of the monoacyl chlorides containing 4-NC and 3,5-DNC could generate limited linear portions within the cross-linked polyamide active layer of the membranes [27]. This can be attributed to the fact that the acid chloride of 4-NC and 3,5-DNC can react with the amino groups of the m-phenylenediamine during interfacial polymerization, resulting in certain uncross-linked regions in the aromatic cross-linked network of the polyamide (Figure 2b,c). This could result in slightly reduced rejection of the salts, but it adds certain important features of increased water permeability and ease of active layer removal for the sake of reuse as low-pressure seawater pretreatment.

To get further confirmatory evidence in favor of the reaction of 4-NC and 3,5-DNC with MPD acting as capping agents during IP, liquid ^1^H NMR of the products obtained was carried out as given in Figure S1a,b. The organic phase was composed solely of the 4-NC or 3,5-DNC, whereas the aqueous phase contained MPD. The confirmatory peak was the NH peak of the amide linkage CONH located at ~10.58 ppm, owing to the reaction between MPD and 4-NC/3,5-DNC. The peaks in the aromatic region from ~7 to 9.5 ppm can be attributed to the aromatic protons owing to the benzene rings of MPD and 4-NC or 3,5-DNC. The existence of the amide peak along with aromatic peaks confirmed the success of the reaction between 4-NC or 3,5-DNC and MPD. Hence, this can act as capping agents in certain sites where the reaction takes place, resulting in slightly lower crosslinking of the 4-NC- or 3,5-DNC-containing membranes. Going further in this dimension, the solid ^13^C NMR of the polyamide active layers of M2 and M3 membranes is given in Figure S2a and S2b, respectively. The presence of all the characteristic peaks was confirmed by the solid NMR, where the carbonyl peaks of the amide linkages of the reaction, primarily between TMC and MPD, as well as the reaction between 4-NC or 3,5-DNC and MPD, were observed at around ~200 ppm. The other peaks, especially the aromatic peaks, were attributed to the benzene rings of the TMC, MPD, and 4-NC or 3,5-DNC. These peaks were found to be located at around 140 to 180 ppm. Owing to the strong electron-withdrawing nature of NO_2_, the peaks were found in the range of 160 to 180 ppm. Upon comparing the spectra of both active layers, there was a slight change in the peaks of this region, which can be attributed to the different chemical environments of the active layers of the membranes.

**Figure 3:**
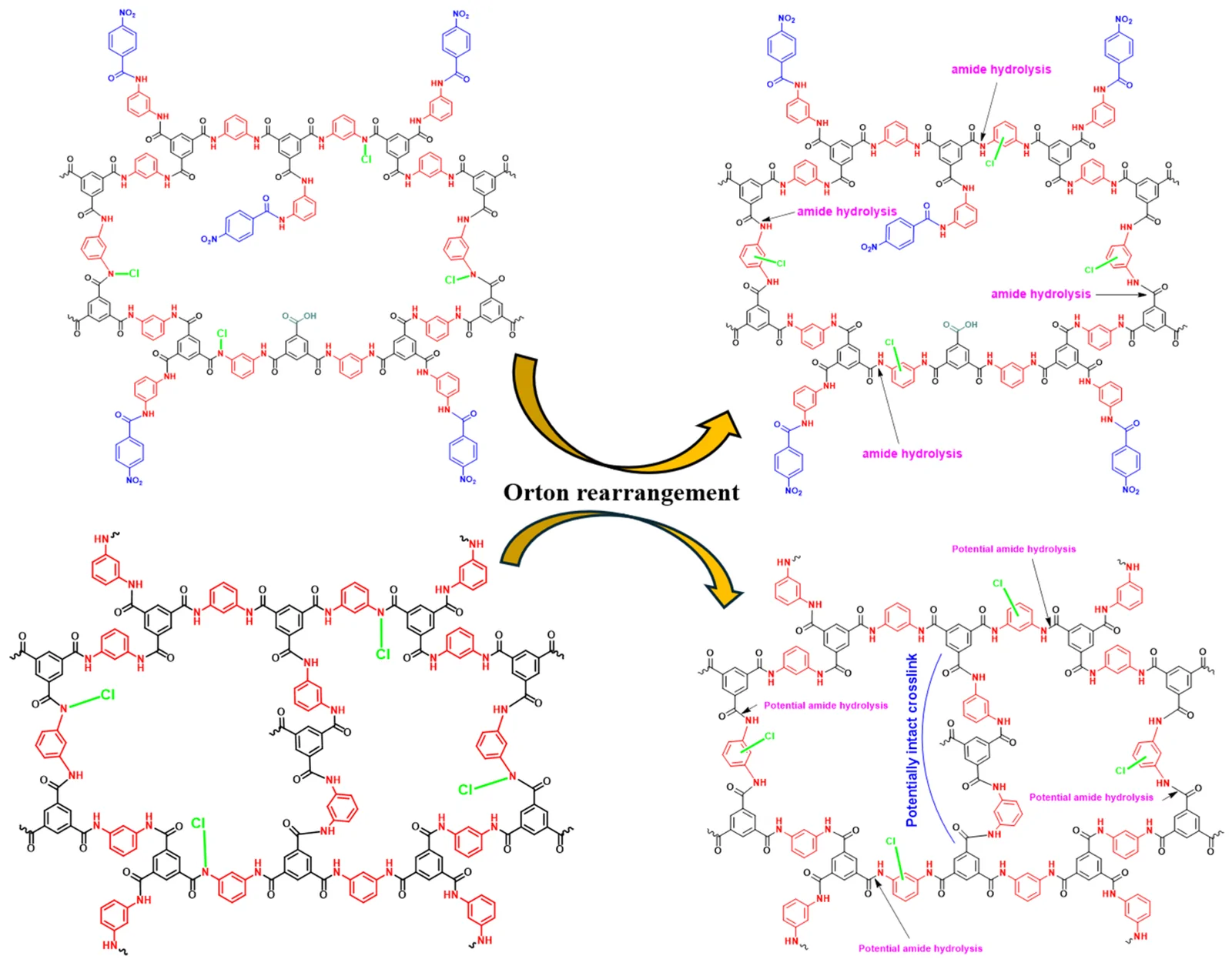
Possible route for the comparative Orton rearrangement and degradation of the aromatic polyamide control m-phenylenediamine and trimesoyl chloride, and m-phenylenediamine with trimesoyl chloride + 4-NC.

The aromatic amide linkage found in the active layer is well known to degrade in the presence of chlorine in the feed [28]. Several studies have explored the possibility of degradation of the amide linkages in the active layer via Orton rearrangement, in which Cl is bonded to the amide N-H, forming N-Cl; in the next step, the Cl is transferred to the aromatic rings of the polyamide network [29,30]. The presence of the electronegative Cl atom weakens the amide linkage and makes it prone to hydrolysis, leading to the breakdown of the polyamide active layer [31]. Figure 3 shows a demonstration of using the same concentration of chlorine for degrading the active layer, where four chlorine atoms have been used as a model for the breaking of the amide linkages of the active layers fabricated in the current study. The ^1^H NMR of the 4-NC or 3,5-DNC reaction with MPD confirmed the successful reaction between the aqueous meta-Phenylenediamine and the organic phase containing 4-NC or 3,5-DNC. Hence, the reaction of the 4-NC or 3,5-DNC with MPD during IP reaction can cap the growing ends of the polyamide active layer at certain points based on the content of the 4-NC or 3,5-DNC in the organic phase. Hence, the additional cross-linking in the case of the control membrane reduces the chances of degradation and makes its reuse a bit challenging in seawater pretreatment or wastewater pretreatment [32]. On the other hand, the active layer of having 4-NC and 3,5-DNC can degrade and be reused for applications in treating wastewater and seawater pretreatment. This would also reduce the environmental and economic challenges associated with EoL SWRO membranes.

**Figure 4:**
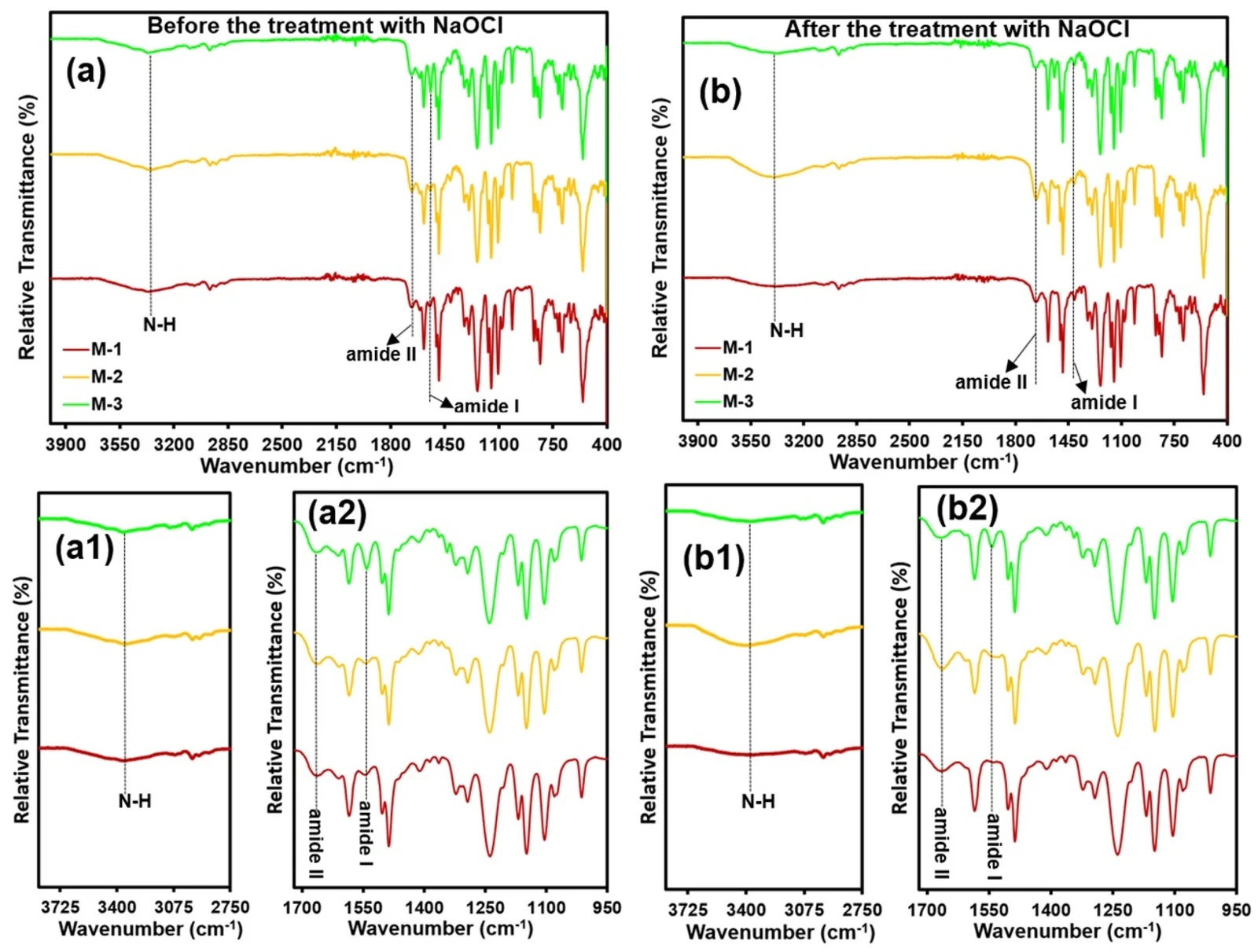
ATR-FTIR spectra of the membranes M1, M2, and M3 before (**a**,**a1**,**a2**), and (**b**,**b1**,**b2**) after exposure to the chlorine solution.

To understand the structural changes in the chemistry of the active layer upon exposure to the NaOCl solution, ATR-FTIR was recorded as shown in Figure 4. Upon comparing the ATR-FTIR spectra of the membranes before (Figure 4a) and after (Figure 4b and Figure S3), the overall spectra seem to be roughly similar to each other. In the case of all membranes, there are distinct peaks attributed to the presence of the functional groups in the active layer, such as the broad peak in the region of ~3350 cm^−1^, which is generally attributed to the N-H stretching vibration of the amide linkage (CONH). The other important peaks confirming the amide linkage were also observed in the region of around 1664 to 1542 cm^−1^, which can be attributed to the amide I and II, respectively [33]. Figure 4b shows that the membranes still possess broad peaks in the region of ~3400 cm^−1^ even after exposure to the solution of NaOCl, which can be attributed to the fact that the polyamide active layer still exists on the polysulfone support. However, the peaks attributed to the amide I and II showed certain variations, such as these peaks showed decline in their intensities upon exposure to NaOCl. This variation is well anticipated and supported by the previously reported evidence of such variations to the polyamide active layer upon exposure to the chlorine solution [34]. Figure 4(a1,a2) show the zoomed-in regions of the prominent peaks of the membranes before exposure to chlorine, and Figure 4(b1,b2) and Figure S3 show the enlarged ATR-FTIR peaks of the membranes after exposure to the chlorine solution. These changes are confirmatory evidence that the membrane-active layers have been degraded. Upon comparing the extent of changes in the active layers of the membranes, the M3 membrane showed a greater decrease in the intensity of the N-H bending vibration, suggesting that the M3 membrane has undergone more drastic changes compared to the other two membranes.

**Figure 5:**
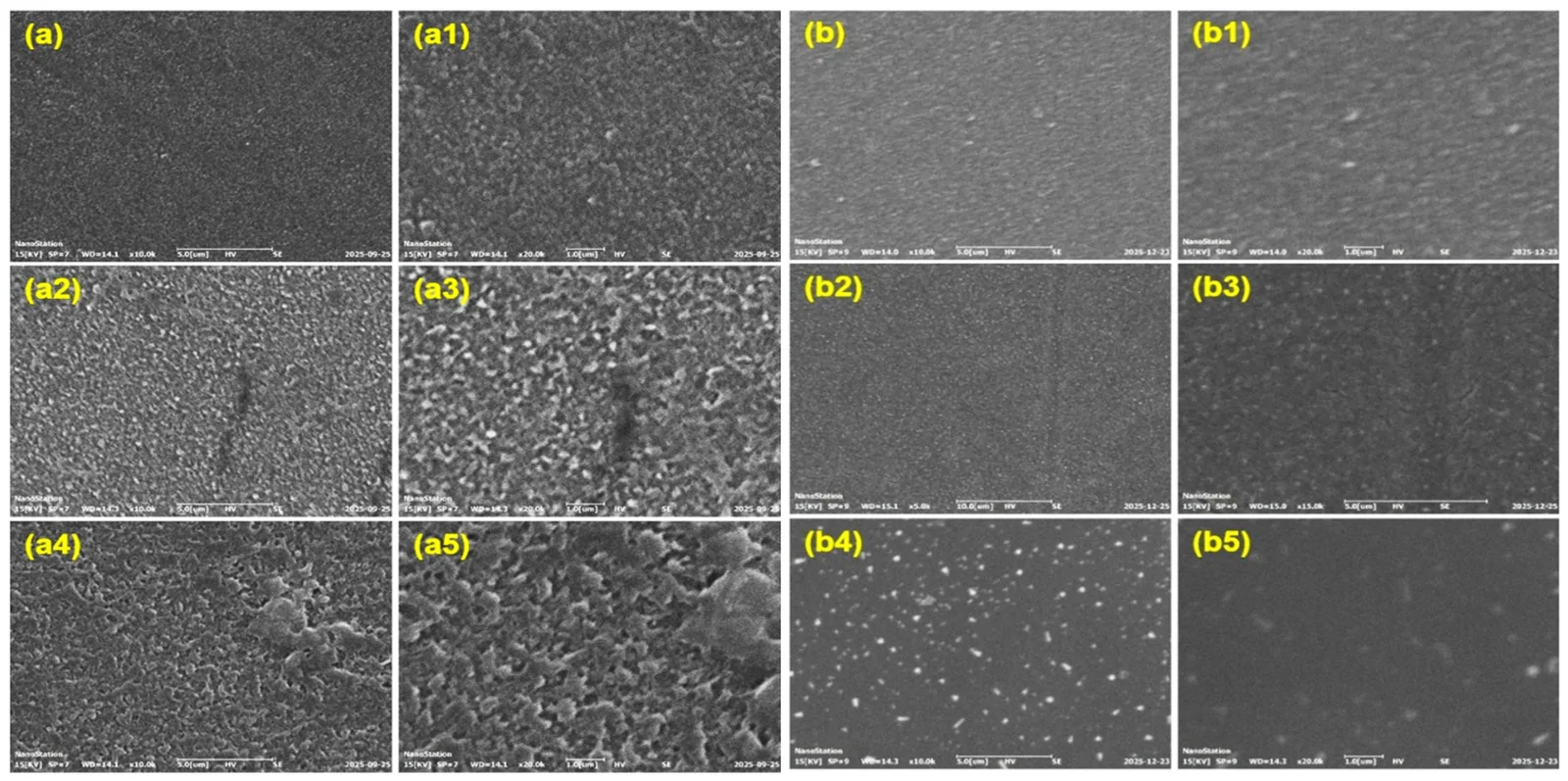
Changes in the surface morphologies of the membranes M1, M2, and M3 before (**a**,**a1**–**a5**) and after (**b**,**b1**–**b5**) exposure to the NaOCl solution.

Another highly important confirmatory piece of evidence was obtained from surface morphological studies of the membranes before and after exposure to chlorine. In the case of M1, M2, and M3 membranes, a distinct polyamide active layer can be seen, with certain variations in the morphologies of the membranes. The polyamide active layer of the control M1 showed the presence of smaller-sized nodules having an appearance of stripes (Figure 5(a,a1)). In the case of the M2 membrane (Figure 5(a2,a3)), these polyamide stripes appeared to be slightly bigger compared to those observed in the case of the M1 membrane. Going further to the M3 membrane (Figure 5(a4,a5)), the polyamide appeared to have even more and bigger globules and stripes. These changes in the surface morphologies of the membranes can be attributed to the changes in the composition of the organic phase used during interfacial polymerization, resulting in changes in the way the polyamide is grown. The presence of the monoacyl chlorinated nitro-substituted additives (4-NC and 3,5-DNC) during interfacial polymerization could react with the amino groups of the m-phenyldiamine and, hence, can cap the growing polyamide chain. Hence, the growing ends of the polyamide active layer can adopt different routes, yielding different morphologies that are possibly observed in the surface SEM micrographs of the membranes. Highly important observations were seen in the case of the membranes after exposure to the solution of chlorine. All membranes showed a significant variation in the surface morphologies owing to the impact of chlorine on degrading the polyamide-active layers of the membranes. Upon comparing surface morphologies of the membranes, it was observed that the polyamide nodules and stripes were significantly diminished in the case of all membranes. Upon close comparison, the membranes, especially M3 (Figure 5(b,b1)), showed a more drastic change in surface morphology, followed by M2 (Figure 5(b2,b3)), while M1 (Figure 5(b4,b5)) showed a slightly less drastic alteration in the membrane. This difference in the apparently different response to NaOCl of the polyamide-active layer highlighted the fact that the polyamide-active layers have different structural features. Another important observation from the SEM micrograph was the presence of a certain amount of the polyamide active layer after exposure to the NaOCl, which can assist in increased rejection of the pollutants with enhanced permeability. Hence, the membrane, such as M3, can be reused for seawater pre-treatment or wastewater treatment, resulting in efficient management of EoL polyamide-desalination membranes and contributing to environmental protection.

**Figure 6:**
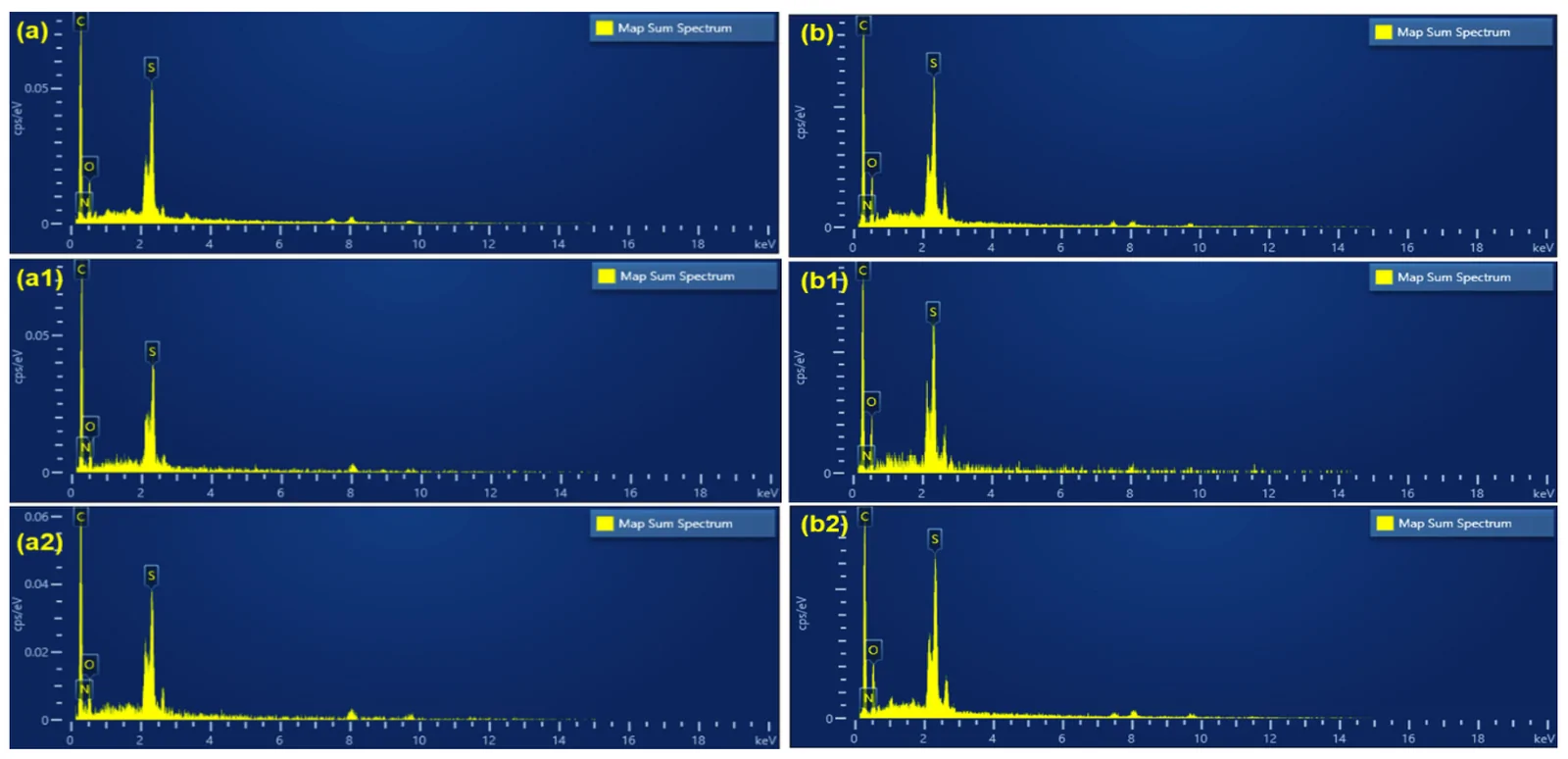
Changes in the chemical composition of the membranes M1, M2, and M3 before (**a**) and (**a1**,**a2**), respectively and after (**b**) and (**b1**,**b2**), respectively exposure to the NaOCl solution.

The changes in the chemical composition of the membranes before and after exposure to the NaOCl solution showed that all membranes underwent certain obvious changes. In the case of all membranes, such as M1 (Figure 6a), M2 (Figure 6(a1)), and M3 (Figure 6(a2)), elements such as C, O, N, and S were identified. These elements are attributed to the polyamide-active layer composed of C, N, and O, whereas S originates from the underlying polysulfone support. In the case of membranes after exposure to NaOCl, M1 (Figure 6b), M2 (Figure 6(b1)), and M3 (Figure 6(b2)), the peak of N was decreased to a certain extent, whereas the peaks of S and O were increased in their intensities, which can be attributed to the degradation of the polyamide-active layer and washing out of the degraded polyamide chains. Moreover, the potential washing out of the polyamide chains could have resulted in exposure of the underlying support composed of polysulfone, leading to increased S and O content. The details of the EDX compositions of the membranes before and after treatment of the membranes with NaOCl are given in Table S1.

**Figure 7:**
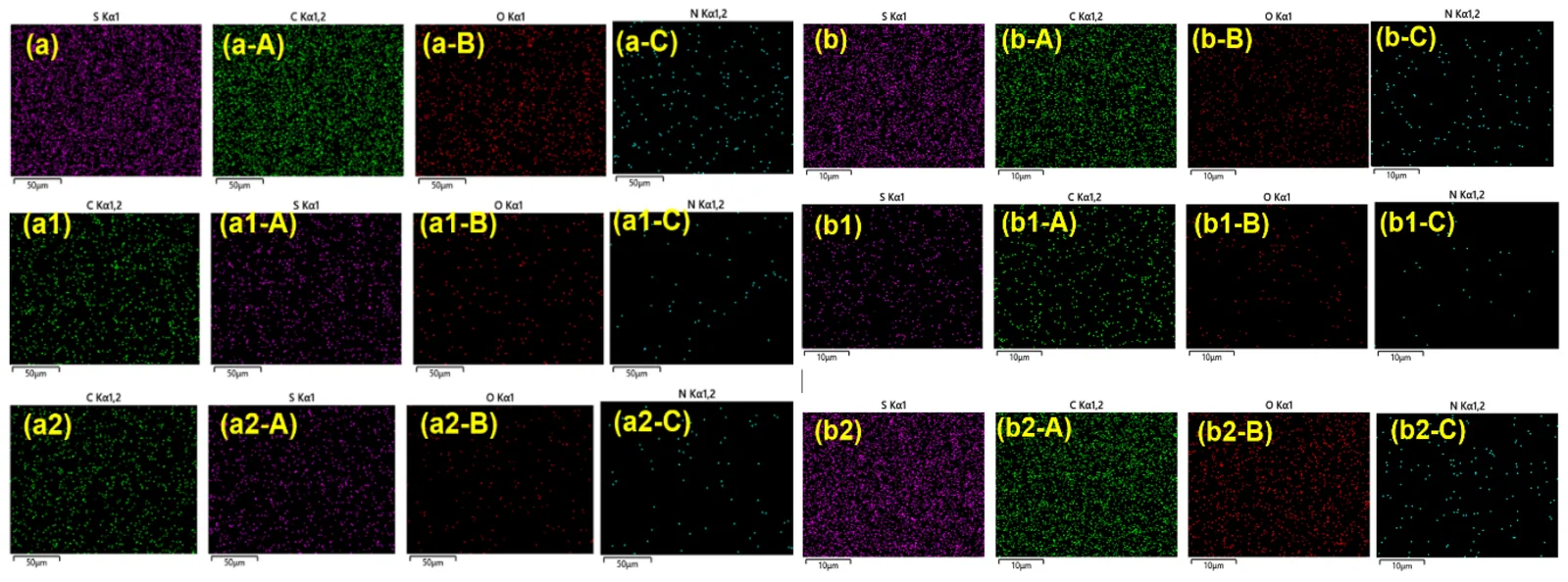
Elemental mapping analyses of the membranes M1 (**a**,**a-A**–**a-C**), M2 (**a1**–**a1-C**), and M3 (**a2**–**a2-C**) before and after exposure to the NaOCl solution of the M1 (**b**,**b-A**–**b-C**), M2 (**b1**–**b1-C**), and M3 (**b2**–**b2-C**).

The corresponding elemental distribution in the scanned sample showed that the elements are present at all locations. Elements such as S, C, O, and N were found to be equally present in the samples of the M1 (Figure 7(a–a-C)), M2 (Figure 7(a1–a1-C)), and M3 (Figure 7(a2–a2-C)) membranes. With the exposure of membranes to NaOCl M1 (Figure 7(b–b-C)), M2 (Figure 7(b1–b1-C)), and M3 (Figure 7(b2–b2-C)), all elements, including S, C, O, and N, were identified, which confirmed the fact that the polyamide-active layer is still present to a certain degree on the polysulfone support.

**Figure 8:**
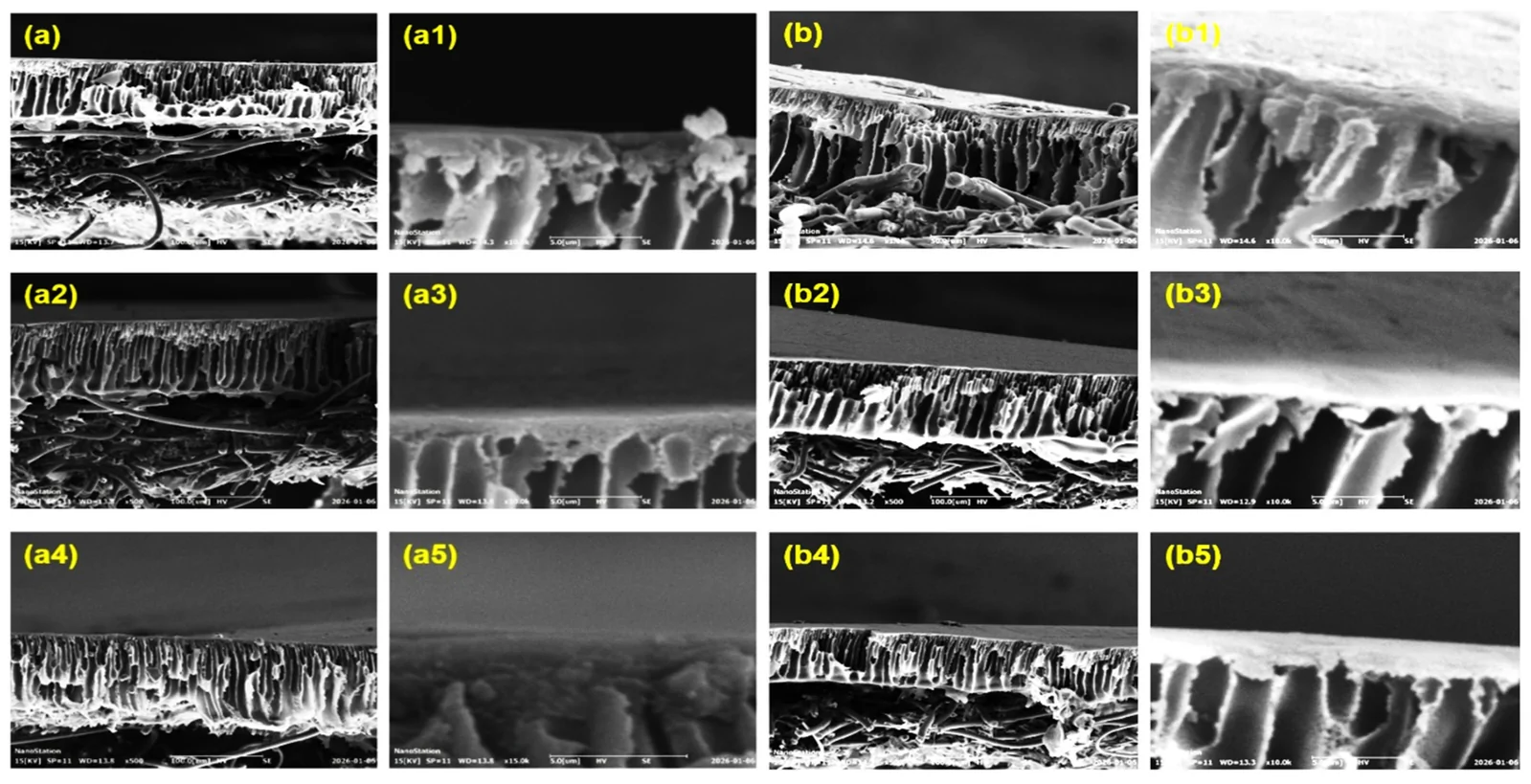
Cross-sectional micrographs of the M1 (**a**,**a1**), M2 (**a2**,**a3**), and M3 (**a4**,**a5**) before NaOCl and after exposure to the NaOCl solution of the M1 (**b**,**b1**), M2 (**b2**,**b3**), and M3 (**b4**,**b5**).

Another important feature of the membranes to determine the effect of the NaOCl was the comparison of the cross-sectional micrographs, where the polyamide-active layer underwent significant degradation. Figure 8(a,a1) show the polyamide-active layer of the M1 control membrane; M2 is shown in Figure 8(a2,a3), whereas the cross-sectional micrographs of the M3 membrane are given in Figure 8(a4,a5). In the case of all the membranes, the presence of the polyamide-active layer can be clearly seen to have significant growth as a dense layer on the underlying finger-like projections of the polysulfone, especially in the case of high-resolution images of the M1 (Figure 8(a1)), M2 (Figure 8(a3)), and M3 (Figure 8(a5)) membranes. The active layer thickness of the membranes was found to be in the range of ~300, 210, and 258 nm for M1, M2, and M3 membranes, respectively. Another observation from these micrographs can be observed from these micrographs is the difference in the growth of the active layers, which can be attributed to the difference in the organic phases used in the fabrication of the membrane. Upon exposure to the NaOCl solution, the polyamide active layer showed a significant decrease in its thickness in the case of all the membranes. (Figure 8(b,b1)) show the active layer variations after exposure to the chlorine solution, where the membrane active layer appeared to have reduced in its thickness to a certain extent. A significant effect on the active layer was seen in the case of the M2 (Figure 8(b2,b3)) and M3 (Figure 8(b4,b5)) membranes. The M1 membrane showed a ~211 nm thickness of the active layer upon exposure to NaOCl. The active layer became quite thin, and this effect was more pronounced in the case of the M3 membrane, where the originally thick polyamide layer was significantly reduced in thickness, as can be seen in the high-resolution micrograph of M3 (Figure 8(b5)). Furthermore, the underlying finger-like projections also showed the absence of the polyamide penetration, as was observed in the cross-sections of the membranes before exposure to chlorine solution. These features are ideal for increased water permeability and removing the large-sized pollutants from the feed water, such as wastewater or seawater. These changes in the structure of the membranes, especially M2 and M3, have revealed the fact that the addition of a reactive additive in the active layer of the membrane has resulted in making the membrane usable for desalination, followed by easy tuning for reuse in applications for wastewater treatment or seawater pretreatment.

**Figure 9:**
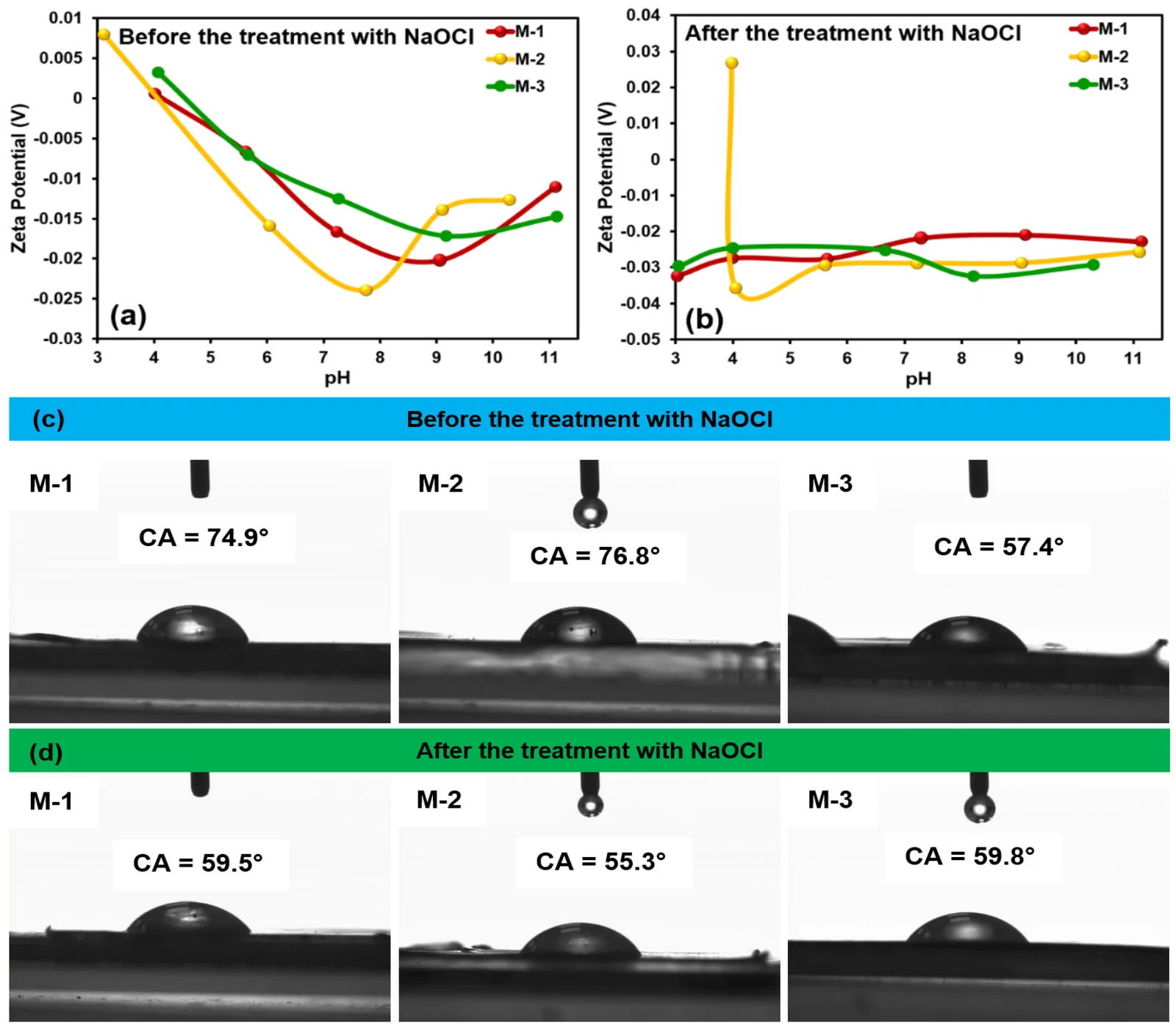
Changes in the zeta-potentials of the membranes (**a**) before, (**b**) after exposure to the NaOCl, changes in the water contact angles of the membranes (**c**) before, and (**d**) after exposure to the NaOCl solution of the M1, M2, and M3.

All of the membranes showed a nearly similar trend in the case of changes in the surface charge of the membranes in response to the changes in the pH of the medium. Figure 9a shows that the membranes showed a positive surface in highly acidic pH, which gradually decreased and changed into a negative surface charge with an increase in the pH of the medium. A further increase in the pH to 10 and 11 showed a slight increase in the surface charge, where the membranes became less negative. This increase in the charge of the membranes at higher pH is also supported by the reported literature, suggesting the accumulation of counter ions such as Na+ ions on the membrane surface at elevated pH [35]. The initial decrease in the surface charge of the membranes can be attributed to the deprotonation of the ammonium ion-containing groups in the active layer. A further increase in the pH causes a further deprotonation of not only the ammonium ion but also that of COOH, resulting in the generation of the negative charge on the surface of the membranes. Among different observations, the most important observation was the fact that all the membranes possessed a more negatively charged surface in the pH range of 6 to 8, which is a pH range that matches the pH range of the feeds of most scenarios. After exposure of the membranes to the NaOCl solution, a significant change was observed in the surface charges of the membranes, where the surface charge remained predominantly negative over acidic and basic pH ranges (Figure 9b). This change in the surface charges of the membranes can be attributed to the impact of NaOCl on the chemistry of the active layer, where hydrolysis of the amide linkages could generate a significant amount of the negatively charged carboxylate groups. Furthermore, the availability of the free amino groups is also reduced, which further reduces the probability of the development of positive charges on the membrane surfaces. Furthermore, the charge was more negative after treatment of the membranes with NaOCl solution, where before NaOCl treatment, the M1, M2, and M3 membranes possessed surface charges of −0.013, −0.017, and −0.024 V, respectively, in the pH range of 7 to 8.5. However, after the treatment of the membranes with NaOCl, the surface charges were recorded to be −0.022, −0.029, and −0.032 V at the pH range of 7 to 8.5 for the M1, M2, and M3 membranes, respectively. Similar changes in the surface charge of the polyamide membranes upon exposure to chlorine have been reported in the literature [31,36]. Moreover, the membranes with reactive additives in their active layers are more responsive to pH changes compared to the control membrane.

The surface wettability of membranes was also found to vary upon exposure of the membranes to NaOCl. Figure 9c shows that the water-contact angles of the membrane were in the range of hydrophilic wettability, where the water-contact angles were found to be 74.9, 76.8, and 57.4° for the M1, M2, and M3 membranes, respectively. Such water-contact angles have been found in the case of the polyamide membranes in the literature [37]. Upon treating the membranes with NaOCl, the water-contact angles of all the membranes were reduced compared to the untreated membranes. Figure 9d shows that the water-contact angles of the M1, M2, and M3 membranes were found to be 59.5, 55.3, and 59.8°, respectively. This decrease in the water-contact angles of the membranes upon exposure to chlorine can be attributed to the generation of carboxylic groups from the hydrolysis of amide linkages in the membranes’ active layer [38]. These changes in the water-contact angles of the membrane clearly indicated that the membrane chemistry has changed.

**Figure 10:**
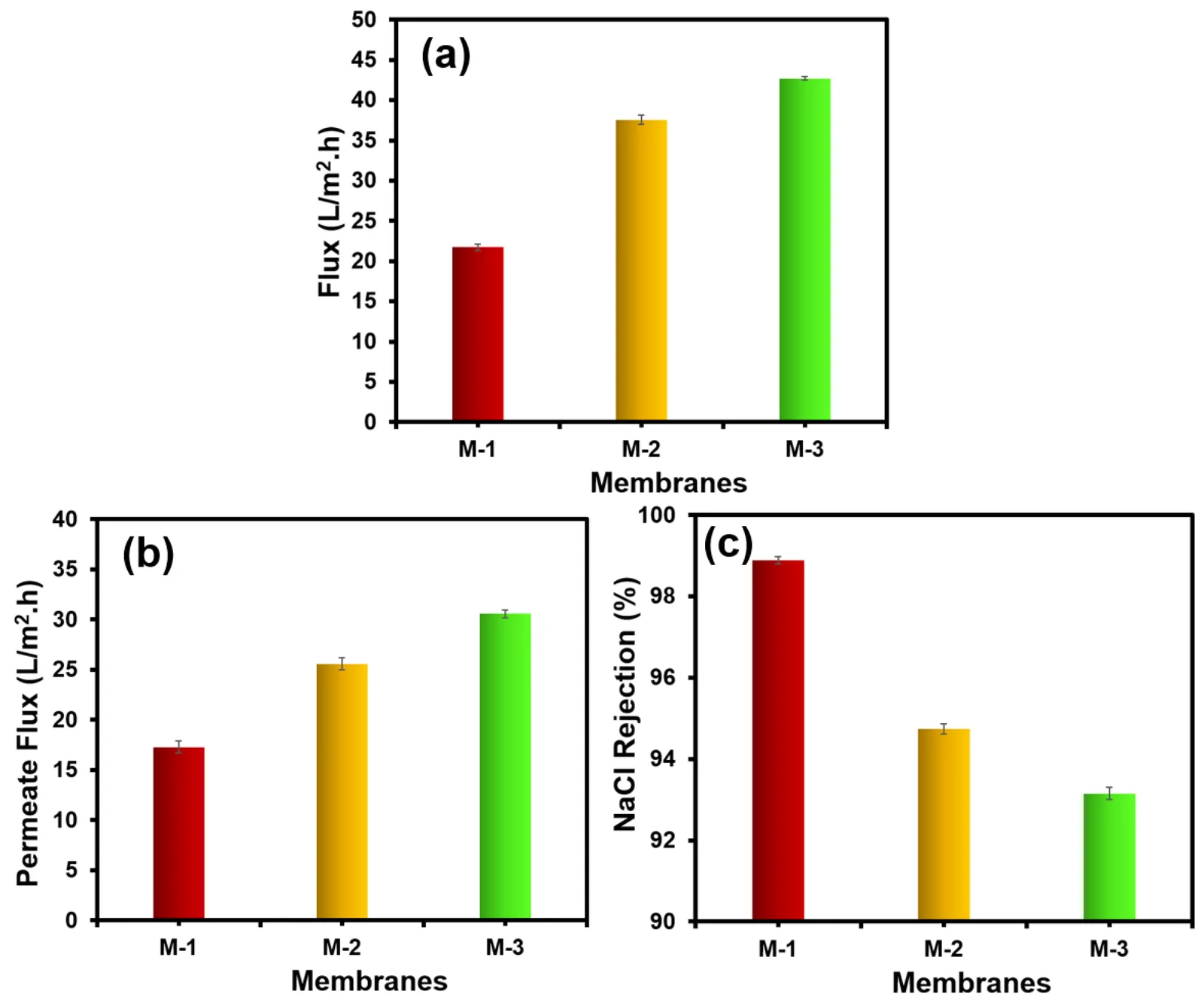
(**a**) Permeate flux of water using distilled water as feed at 30 bar, (**b**) permeate flux, and (**c**) the rejection of NaCl using 1000 ppm NaCl feed at 30 bar in the case of the M1, M2, and M3.

Upon characterization, the membranes were tested for their desalination performance. Figure 10a shows that the permeate flux with distilled water was found to be ~22, ~37, and ~42 L m^−2^ h^−1^ at 30 bar for the M1, M2, and M3 membranes, respectively. The increase in the permeate flux upon addition of the 4-DNC and 3,5-DNC could be attributed to the reaction of these molecules with m-phenylenediamine during interfacial polymerization. This reaction can lead to the generation of some areas of linear polyamides, increasing the size of the permeation channels. The permeate flux was found to be decreased when the salt-containing feed was used during the desalination experiment. The permeate flux was found to be 16.8, 26.0, and 30.2 L m^−2^ h^−1^ with an NaCl-containing feed for the M1, M2, and M3 membranes, respectively (Figure 10b). This can be attributed to the development of a concentration polarization (CP) layer on the surface of the membranes. This CP layer offers resistance to the flow of water through the membrane and increases the resistance to the flow of water through the membrane, causing a decline in permeate flux. In the case of the rejection of NaCl, rejection was also found to vary from ~99% to 94% and 93% in the case of M1, M2, and M3 membranes, respectively (Figure 10c). It was observed that the membranes with high salt rejection, such as M1, have low permeate flux, which can be attributed to the well-known permeability–selectivity tradeoff. Similar behavior can also be observed for the M2 and M3 membranes as well.

**Figure 11:**
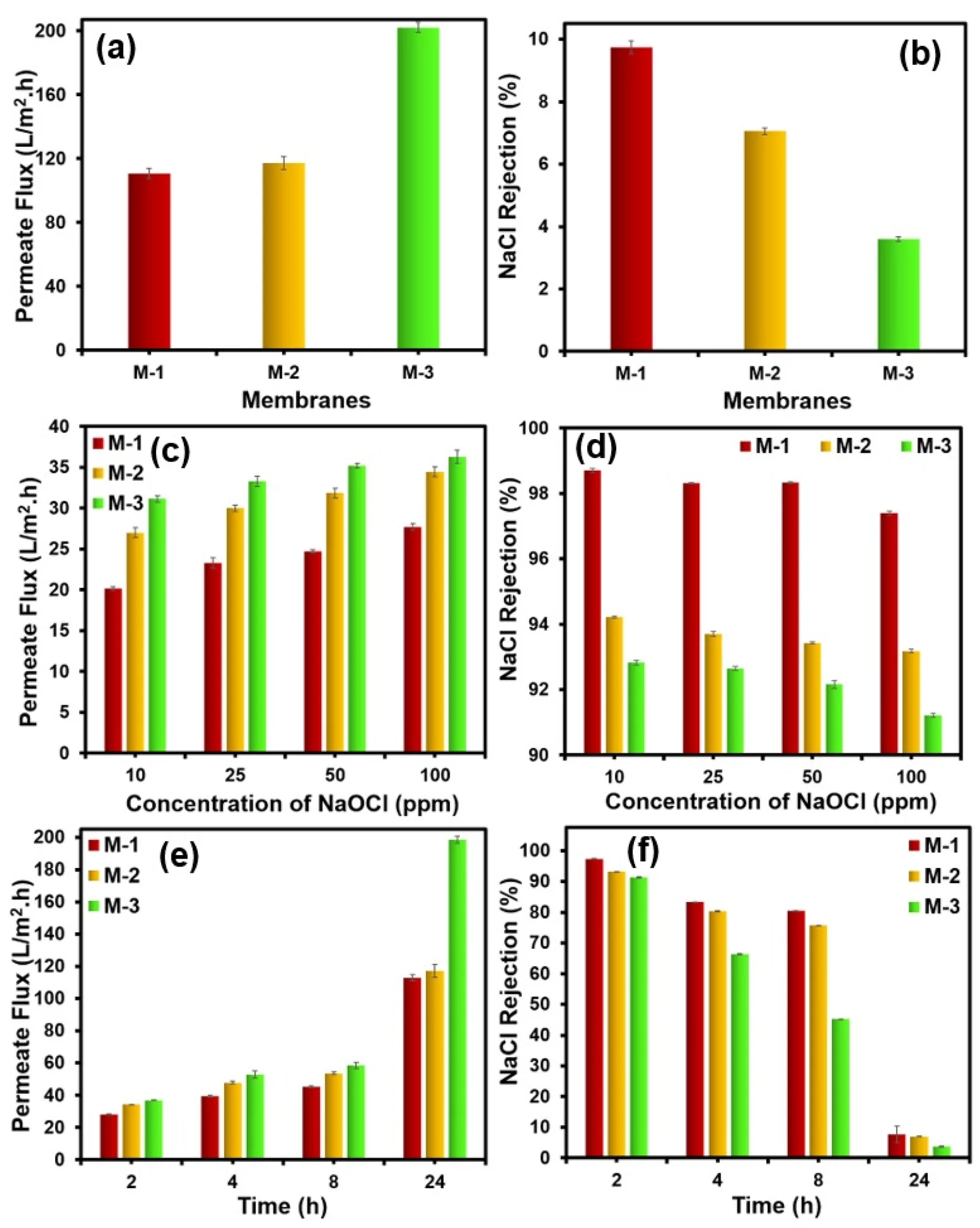
(**a**) Permeate flux, and (**b**) rejection of NaCl using 10,000 ppm NaCl feed at a feed pressure of 30 bar using 100 ppm NaOCl feed after soaking the membranes for 24 h. Effect of increasing NaOCl concentration on the (**c**) permeate flux, and (**d**) rejection of NaCl using 10,000 ppm NaCl feed at a pressure of 30 bar. Effect of increasing time on the (**e**) permeate flux, and (**f**) rejection of NaCl using 10,000 ppm feed, 100 ppm NaOCl concentration, and a feed pressure of 30 bar in the case of the M1, M2, and M3.

The effect of chlorine on the degradation of the polyamide and its effect on permeability and selectivity was investigated for the sake of reuse in pretreatment applications. Different experiments were carried out to explore the behaviour of the membranes towards exposure to chlorine. Figure 11a shows the permeate flux of the membranes after exposure to a chlorine solution for 24 h. The permeate flux was found to be 108, 114, and 200 L m^−2^ h^−1^ for the M1, M2, and M3 membranes, respectively, at a feed pressure of 30 bar. Among the different membranes, the most significant enhancement was observed in the case of the M3 membrane, achieving nearly a 6.5-fold increase in permeate flux using NaCl as feed compared to the M3 membrane before chlorine exposure. This can be attributed to the ease of degradation of the polyamide-active layer in the case of the M3 membrane compared to the control M1 membrane, owing to some uncross-linked plugs of polyamide in the active layer. Figure 11b shows the NaCl rejection by the membranes after 24 h of exposure to the NaOCl solution. The rejection of NaCl significantly dropped to 9.6, 7.1, and 3.5% in the case of M1, M2, and M3 membranes. This experiment confirmed the fact that active layers of the membranes have been degraded to a great extent, allowing more water and NaCl to pass through the membrane. This was further confirmed by the rejection of the divalent salts such as Na_2_SO_4_ by the membranes. Figure S4a shows the rejection of Na_2_SO_4_ to be 62, 28, and 25% by the M1, M2, and M3 membranes, respectively. The M2, and especially the M3, membrane appeared to be more responsive to the chlorine solution and developed features required for seawater pretreatment.

The effect of increasing the dosage of the NaOCl was studied, as shown in Figure 11c, where it was obvious that the increased concentration of NaOCl caused increased damage to the active layer of the membrane. Figure 11d shows that the rejection of NaCl was decreased to ~97, 93, and 91 L m^−2^ h^−1^ in the case of M1, M2, and M3 membranes. This degradation was not enough to reach high permeability since the degradation was not significant using 100 ppm of NaOCl for 2 h of testing. To reach a further degradation of the polyamide-active layer, the time of contact of the NaOCl was increased. Figure 11e shows that the permeate flux using NaCl feed increased from 28 to 108, 34 to 114, and 36 to 200 L m^−2^ h^−1^ as the contact time of the NaOCl was increased from 2 to 24 h in the case of M1, M2, and M3 membranes, respectively. As the permeate flux was increased with the increase in the contact time of the NaOCl, the rejection of NaCl was expected to decrease. Figure 11f shows that the rejection of the NaCl was decreased from 97 to 9.6, 93 to 7.1, and 91 to 3.6% with an increase in contact time from 2 h to 24 h for M1, M2, and M3 membranes, respectively. These changes in the active layer of the membranes reflected that the membranes with the presence of the reactive additives in the active layer resulted in enhanced degradation compared to the control membrane. Such membranes can be readily reused after EoL for applications in seawater and wastewater treatment.

**Figure 12:**
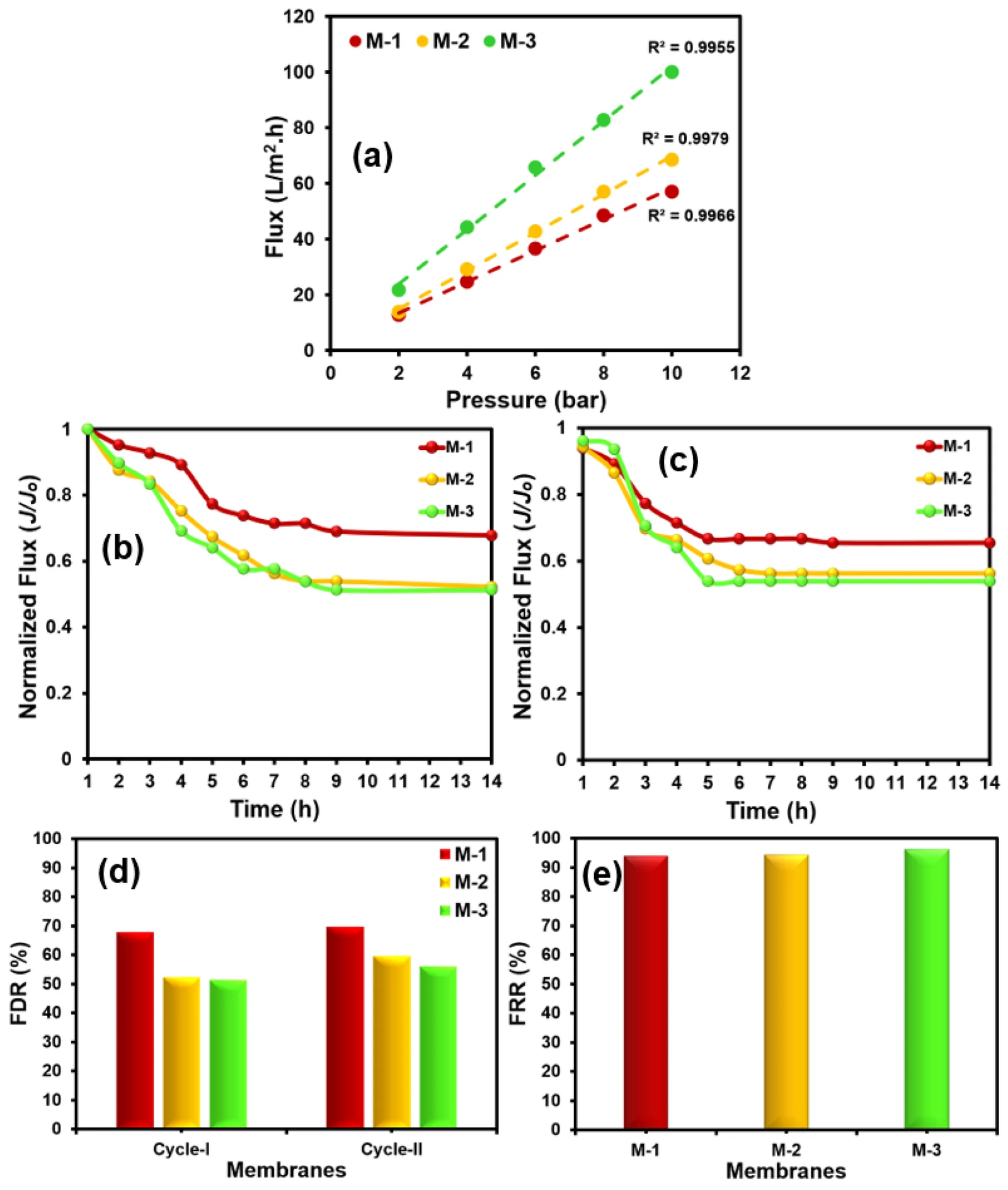
(**a**) Effect of increasing feed pressure on the permeate flux using 100 ppm BSA + 1000 ppm NaCl as feed. Fouling studies of the membranes in terms of variations in normalized flux during (**b**) 1st and (**c**) 2nd cycle of fouling for a duration of 14 h using BSA + NaCl as feed at a feed pressure of 4 bar and (**d**) FDR and (**e**) FRR of the membranes following the fouling and cleaning phase for the M1, M2, and M3 by flushing the membranes with water for a duration of 30 min.

To study the pretreatment potential of the membranes for rejection of the model foulants mimicking the foulants generally found in seawater/wastewater, the M1, M2, and M3 membranes were applied for the rejection of BSA, sodium alginate (SA), and humic acid (HA). The membranes showed excellent rejection of these foulants in the range of >97 to >99%, as shown in Figure S4b. This shows that the membranes after treatment with NaOCl have lost their desalination potential for divalent and monovalent salts, which was also confirmed by the molecular weight cut-off (MWCO) measurements of the membranes. The MWCOs of the membranes were found to be more than 1000 Da (Figure S5), which suggested that the membranes were not in the NF range. This further suggested that the NaOCl treated membranes lie in the UF range, which can be used for pretreatment applications. Following this study, BSA was studied further for the fouling performance of the NaOCl membranes, where BSA was used as a model foulant in the NaCl-containing feed. Figure 12a shows that permeate flux increased with an increase in the feed pressure, with an increase from ~12 to 57, 14 to 68, and 21 to 100 L m^−2^ h^−1^ when the feed pressure was increased from 2 to 10 bar for M1, M2, and M3 membranes, respectively. However, it is important to observe that the permeate flux was nearly reduced to half when BSA was used with NaCl compared to the use of only NaCl as feed. This observation suggested that the presence of the charged foulants in the feed could result in developing interactions with the charged groups in the membrane active layer of the membranes. These interactions might include hydrogen bonding and electrostatic interactions between the foulants and the membrane surfaces. The action of chlorine is known to create groups such as -COOH and NH-Cl groups owing to hydrolysis of the amide linkage. The next experiment explored the fouling behaviors of the membranes using BSA and NaCl as feed for a duration of continuous cross-flow operation for 14 h. Figure 12b shows a decline in normalized values with increasing time of testing the membrane, with a more obvious decline during the initial 6 h of operation, where the normalized flux reached a value of 0.57 and eventually stabilized at a value of ~0.51 until the testing duration of 14 h in the case of the M3. The control M1 membrane showed a slightly better antifouling performance, which might be attributed to the smaller number of exposed groups on the membrane owing to hydrolysis of the polyamide active layer. The initial decline in the normalized flux is due to the possibility of attaching more foulants to the binding sites on the surface of the membrane. These sites can eventually be covered by the attachment of foulants to the membrane surface, and hence the normalized flux was stabilized after ~6 h of testing the membranes with BSA. There is no doubt that fouling is unavoidable despite the ideal chemistry and surface features of the membranes. Therefore, the important aspect of the membranes is the recovery of the permeate flux upon cleaning. The membranes were cleaned by flushing the surface of the membranes with distilled water for 30 min, during which the membranes showed excellent recovery of their permeate flux following the cleaning cycle. Figure 12c shows that the M3 membrane showed a slightly better recovery of the normalized flux, reaching a value of 0.96 compared to 0.94 for the other two membranes. However, the decline of the normalized flux upon fouling happened at nearly the same time for all the membranes, where the M3 showed slightly more decline. However, it is important to mention here that even though M3 showed comparatively more decline in normalized flux, the overall flux of the M3 membrane was significantly higher compared to the other membranes. The flux decline ratio (FDR) and flux recovery ratios (FRR) of the membranes are depicted in Figure 12d,e for the first and second cycles of cleaning the membranes, respectively. The tailored membranes showed more decline in permeate flux compared to the other control membrane, which might be the result of the increased number of binding sites available on these membranes after exposure to the chlorine solution. During the first cycle of fouling, the M2 and M3 membranes showed a decline of ~50%, which was found to be 40% during the second cycle of fouling. It is important to note that the FRR of the tuned membranes was more than that of the control membrane, which suggested that the fouling was reversible and was removed on cleaning with water. Although the current strategy showed the enhanced sensitivity of the tailored membranes M2 and M3 to the exposure of NaOCl, the detailed study under real-life conditions still needs to be explored.

## 4. Conclusions

This study demonstrated that a slight modification in the active layer chemistry of the membrane can result in highly chlorine-sensitive membranes. These polyamide membranes could be recycled by degrading the active polyamide layers of the membranes to a smaller quantity of chlorine compared to the control membranes, which require extended chlorine-exposure intensity. The monobenzoyl-chlorinated additives 4-nitrobenzoyl chloride (4-NC) and 3,5-dinitrobenzoyl chloride (3,5-DNC) were added to the organic phase during interfacial polymerization. Since the 4-NC and 3,5-DNC possess only one reactive site compared to three reaction sites on trimesoyl chloride, the 4-NC and 3,5-DNC can act as capping agents for certain chains in the crosslinked polyamide active layer of the membrane. Hence, a limited number of uncross-linked regions can be generated, which could potentially increase the vulnerability of the polyamide-active layer of the membranes to the low-intensity exposure of 100 ppm NaOCl for 24 h. The permeate fluxes of the M1, M2, and M3 were found to be ~22, ~37, and ~42 L m^−2^ h^−1^ at 30 bar before exposure to NaOCl solution. These were increased to 108, 114, and 200 L m^−2^ h^−1^ in the case of M1, M2, and M3 membranes after exposure to 100 ppm of NaOCl for 24 h.

## Data Availability

The original contributions presented in this study are included in the article/Supplementary Material. Further inquiries can be directed to the corresponding author.

## Supplementary Materials

The following supporting information can be downloaded at: https://www.mdpi.com/article/10.3390/polym18172153/s1.
