# Supplementary material for: Can Tailoring the Polyamide Active Layer of the Desalination Membranes Ease the Recycling of End-of-Life Membranes for Seawater Pretreatment Applications
Source: Polymers (Basel). 2026 Sep 3;18(17):2153. doi: 10.3390/polym18172153 (PMC13568041; doi:10.3390/polym18172153)
Supplement: Supplementary file 1 [file polymers-18-02153-s001.zip › polymers-4459621-supplementary.pdf]

polymers

MDPI

Supplementary Information File

Can Tailoring the Polyamide Active Layer of the Desalination Membranes Ease the Recycling of End of Life Membranes for Seawater Pretreatment Applications

Abdul Waheed and Umair Baig *

Interdisciplinary Research Center for Membranes and Water Security, King Fahd University of Petroleum & Minerals, Dhahran 31261, Saudi Arabia; abdul.waheed@kfupm.edu.sa
* Correspondence: umairbaig@kfupm.edu.sa

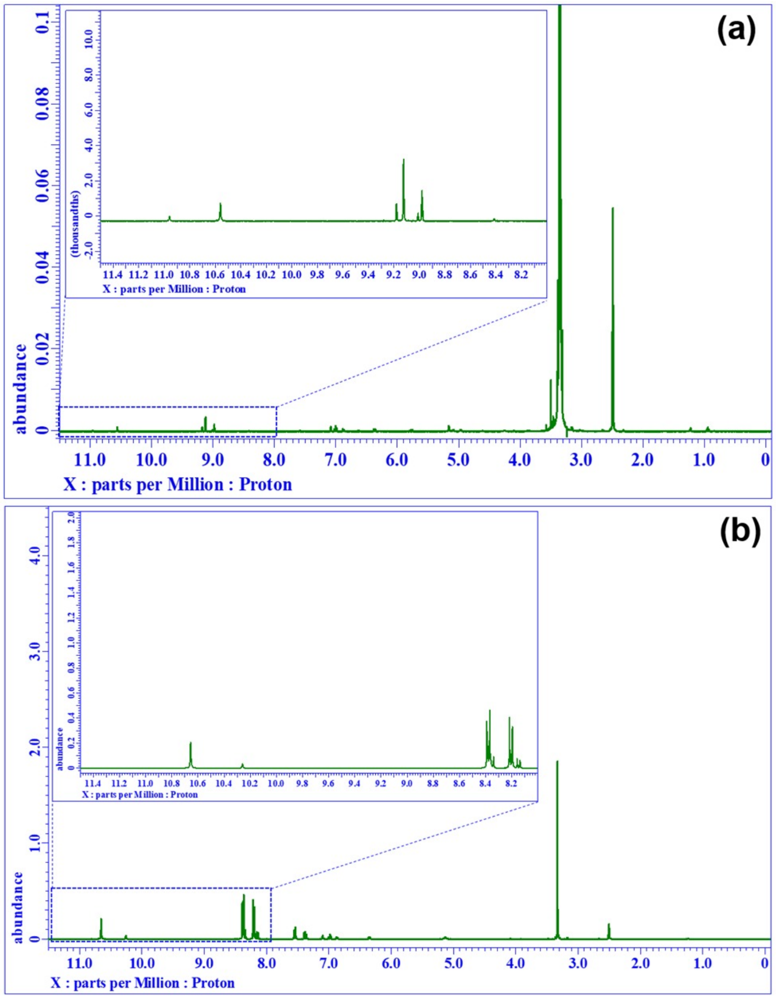

Figure S1. ^1H NMR (DMSO-d_6) of the products obtained from the reaction between MPD and (a) 4-NC and (b) 3,5-DNC. The products were obtained by dissolving small amounts of 4-NC or 3,5-DNC in the organic phase without using TMC, and the aqueous phase was composed of MPD.

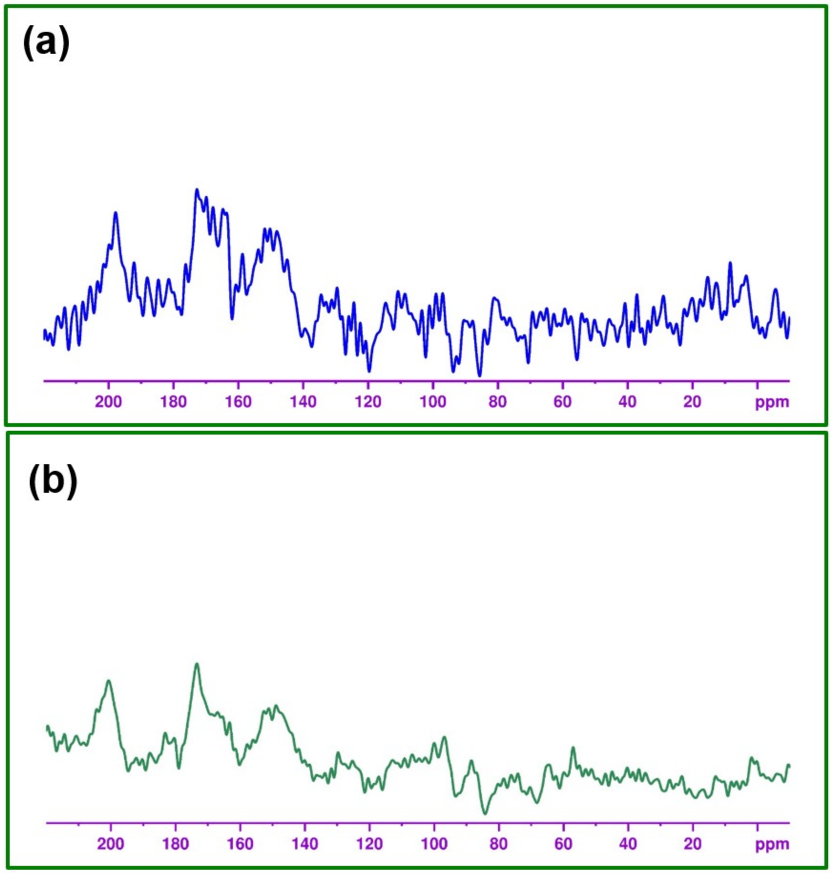

Figure S2. Solid ^{13}C NMR of the polyamide active layers of M2 and M3 membranes.

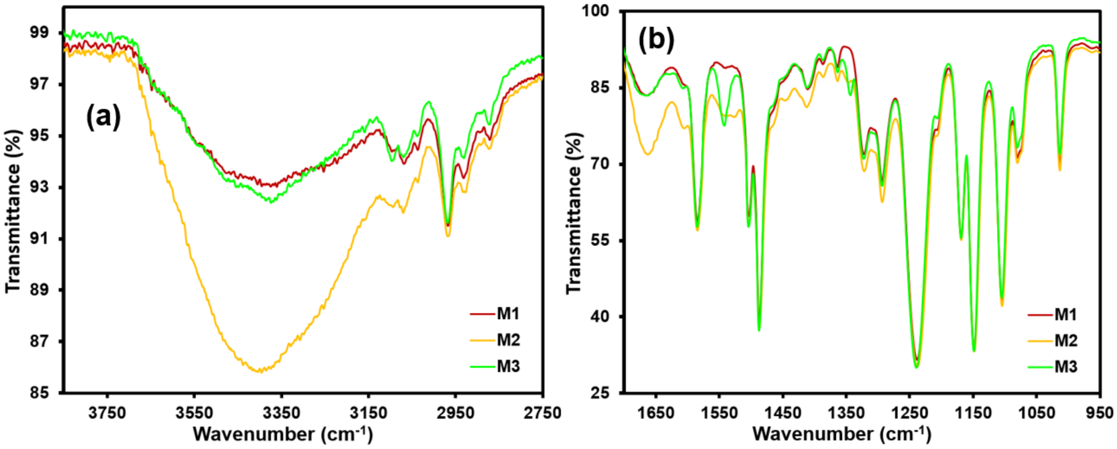

Figure S3. ATR-FTIR spectra of the M1, M2, and M3 membranes after treatment with NaOCl.

http://doi.org/10.3390/polym18172153

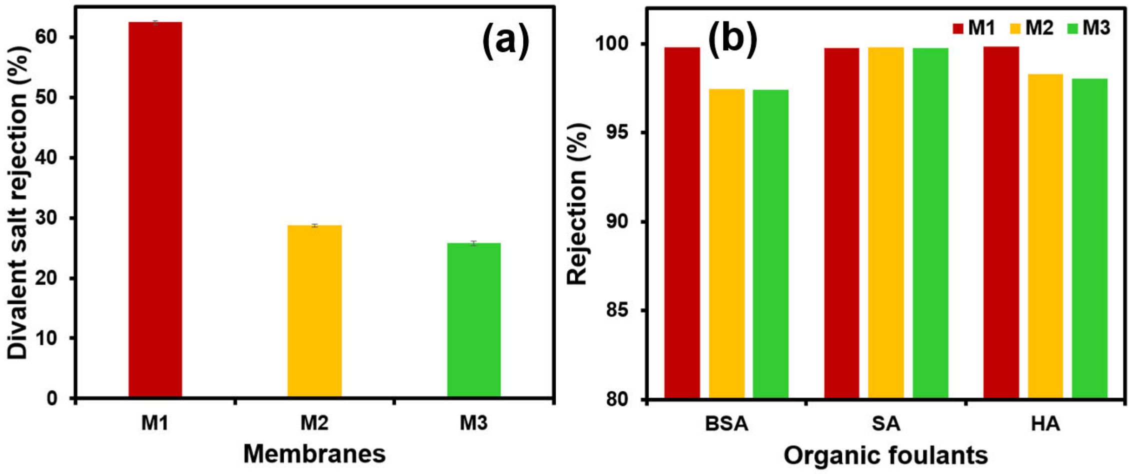

Figure S4. (a) Rejection of Na_2SO_4 as a divalent salt, and (b) rejection of the different model foulants such as BSA, HA, and SA found in the seawater feed by M1, M2, and M3 membranes after NaOCl treatment.

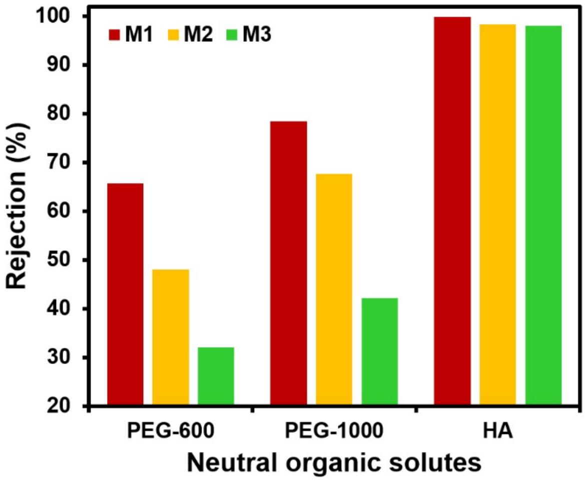

Figure S5. Rejection of neutral molecules of varying size by M1, M2, and M3 membranes after NaOCl treatment.

Table S1. Elemental composition of the membranes before and after exposure to NaOCl.

Membranes Before NaOCl Treatment	Elements/Weight (%)			
	C	N	O	S
**M1**	76.12	6.40	11.98	5.50
**M2**	75.49	3.92	13.73	6.86
**M3**	79.38	2.74	12.26	5.62
**Membranes After NaOCl Treatment**	**Elements**			
	**C**	**N**	**O**	**S**
**M1**	72.15	4.64	16.42	6.79
**M2**	74.27	1.12	17.21	7.41
**M3**	75.09	1.98	15.82	7.11

http://doi.org/10.3390/polym18172153
